# Health Challenges in Vulnerable Populations: Neurological and Vascular Diseases Among People Experiencing Homelessness in Gdańsk, Poland: An Observational Study

**DOI:** 10.3390/jcm15062278

**Published:** 2026-03-17

**Authors:** Krzysztof B. Klimiuk, Michał Błaszczyk-Niezgoda, Anna Kurek, Piotr Glimasiński, Dawid Krefta, Łukasz Balwicki

**Affiliations:** 1 Department of Public Health and Social Medicine, Faculty of Health Sciences, Medical University of Gdańsk, 80-214 Gdańsk, Poland; krzysztof.klimiuk@gumed.edu.pl (K.B.K.); lukasz.balwicki@gumed.edu.pl (Ł.B.); 2 Department of Neurophysiology, Neuropsychology and Neuroinformatics, Faculty of Health Sciences, Medical University of Gdańsk, 80-214 Gdańsk, Poland; 3 Department of Neurology and Stroke, Nicolaus Copernicus Hospital, COPERNICUS PL Sp. z o. o., 80-803 Gdańsk, Poland; 4 Faculty of Medicine, Medical University of Gdańsk, 80-214 Gdańsk, Poland; 5 Gdańsk Branch, Saint Brother Albert’s Aid Society, 80-518 Gdańsk, Poland; 6 Warsaw School of Economics, 02-554 Warsaw, Poland

**Keywords:** homelessness, health inequalities, chronic disease, neurological conditions, public health

## Abstract

**Background/Objectives**: People experiencing homelessness (PEH) face greater morbidity, multimorbidity, and premature mortality than the general population. Medical data on this population in Gdańsk remain scarce. The aim of this study was to assess the prevalence, age distribution, comorbidity burden, and healthcare utilization of selected neurological and vascular diseases among homeless men in Gdańsk, Poland. **Methods**: A retrospective secondary analysis was performed using data from 551 men residing in shelters operated by the largest PEH support organization in Gdańsk. A random sample of 226 individuals (95% confidence level) was analyzed, selected by randomization in Microsoft Excel. Data were extracted from interviews, verified medical documentation, and staff records. **Results**: Mean age was 57.0 (SD 12.9) years (median 60). Among the studied sample, essential (primary) hypertension (20.4%), heart failure (10.2%), atrial fibrillation (8.9%), and chronic obstructive pulmonary disease (8.4%) were the most common conditions. Sequelae of cerebrovascular disease (ICD-10: I69) affected 8.9% of participants; this subgroup was older and had higher rates of disability certification and hospitalization than the overall sample. Epilepsy (12.0%) and polyneuropathy (4.0%) differed in age distribution, disability rates, and comorbidity burden, with the epilepsy subgroup displaying high substance-use prevalence. Overall, 44.0% of the sample had been hospitalized since 2019. **Conclusions**: Homeless men in Gdańsk present a high burden of neurological and vascular disease at comparatively young ages, along with substantial multimorbidity. These findings highlight structural inequalities in healthcare access and the need for integrated, equity-oriented health and social care interventions.

## 1. Introduction

The European Federation of National Organizations Working with the Homeless (FEANTSA) provides a comprehensive definition of homelessness, which includes being roofless, houseless, or living in insecure or inadequate housing [1]. Vulnerable populations, such as people experiencing homelessness (PEH), are particularly exposed to layered, multidimensional forms of exclusion that extend beyond social marginalization into economic precarity and limited access to healthcare services [2]. Homelessness is gaining increasing political visibility in Europe as the costs of housing and living are rising. Limited affordability and accessibility of housing and energy poverty increasingly affect vulnerable populations and the number of PEHs continues to grow [3]. According to a 2024 FEANTSA report, approximately 1,277,000 people are homeless (including rough sleepers, those in shelters, and those in temporary accommodation [3]).

In Poland, every two years, a national count of people experiencing homelessness (PEH) is conducted; the most recent took place during a single night in February 2024. Pomeranian Voivodeship recorded the highest number of individuals suffering from the homelessness crisis in the country [4]. It is one of Poland’s 16 administrative regions, located in the northern part of the country on the southern Baltic coast. At the end of 2024, it had 2,359,493 inhabitants (6.3% of Poland’s population); 62.1% of people lived in the urban areas. In 2023, its gross domestic product (GDP) per capita was 82,600 Polish zlotys (PLN), which was about 95.4% of the national average, ranking 5th in the country, indicating that it is a rather wealthy region [5]. Among a total of 31,042 PEH identified nationwide, 15% were located in this voivodeship. Furthermore, Pomerania is one of four voivodeships where this number has increased in recent years [4]. As Gdańsk is the largest city in Pomerania, addressing homelessness at the local level is of particular importance. In Poland, research on homelessness remains limited, particularly regarding the specific healthcare problems faced by this population. Although various studies have been conducted in certain Polish cities, most have focused on socioeconomic factors and have largely relied on survey- or questionnaire-based methods, which can emphasize subjective experience over medical data [6,7,8]. One notable exception is a study from Bydgoszcz that examined the health of homeless individuals presenting to the emergency department (ED) [9]. However, not all health problems manifest as acute crises requiring ED visits. Other studies based on shelter or hospital data have focused on causes of death and the impact of weather conditions (rather than the prevalence of specific diseases) [10] or alcohol consumption [11].

To the best of our knowledge, no study has provided a comprehensive overview of the health conditions affecting PEH in Gdańsk. While one recent study included some individuals from Gdańsk, its primary focus was on subjective well-being, daily functioning, and socioeconomic factors rather than medical diagnosis, and it was based on self-reported data [6]. The aim of the present study is to assess the prevalence of neurological and vascular diseases among men experiencing homelessness in Gdańsk. This study aims to provide a more accurate and complex understanding of their health status and identify key areas for medical and public health interventions.

## 2. Methods

### 2.1. Overview and Study Design

This study is a continuation of a previously conducted retrospective analysis based on data collected by the Saint Brother Albert’s Aid Society (TPBA), Gdańsk branch [12], the largest non-governmental provider of services for people experiencing homelessness (PEH) in the Trójmiasto metropolitan area. It is responsible for the majority of support in Gdańsk. The city, located in northern Poland, had a population of 487,371 in 2023, with an estimated 1000–1300 homeless individuals [13].

The dataset, originally compiled for operational and support purposes, included health and social information on 551 homeless men residing in TPBA shelters in 2023. The data were collected by social workers, paramedics, nurses, caregivers, and street workers. They comprised social assessments conducted by social workers, health interviews, and the compilation of medical documentation by medical staff, as well as service notes prepared by caregivers and street workers. No independent physical examination was performed for research purposes. The records reflected information routinely collected during service provision, rather than data generated through a dedicated study-specific clinical assessment. As this is a secondary analysis of existing non-experimental data, the Bioethics Committee at the Medical University of Gdańsk confirmed that formal ethical approval was not needed on 5 September 2024.

### 2.2. Dataset

This study is based on a secondary analysis of a dataset previously collected by Saint Brother Albert’s Aid Society (TPBA). From the full population of 551 male residents of the TPBA shelters, a random sample of 226 individuals was selected for analysis. Each resident had been assigned an internal ID during dataset preparation. The study sample was then selected in Microsoft Excel 2022 by random selection of records based on these IDs. The selection ensured a 95% confidence level while respecting operational, ethical, and privacy considerations. The emphasis on male individuals is justified by the fact that men represent approximately 80% of the homeless population in Poland.

The source records included structured health interviews, staff notes, medication information, specialist certificates, hospital discharge summaries, and other available medical documentation contained in the operational files. These materials were reviewed and standardized by a volunteer physician affiliated with TPBA who had access to the full set of available notes for each selected individual. No new diagnoses were established for the purpose of this study; diagnoses were coded according to ICD-10 based on documented medical history available in the files, with the exception of prior ischemic stroke (ICD-10: I63), which—if documented after the acute phase—was coded as I69 (sequelae of cerebrovascular disease).

The dataset included sociodemographic characteristics, health status (including chronic diseases and disability certification), healthcare utilization (including hospitalizations since 2019), and types of assistance received. Healthcare utilization data referred primarily to documented hospitalizations recorded in discharge summaries and administrative notes; however, when formal documentation was unavailable, past hospitalizations could also be recorded on the basis of self-reported information documented by TPBA staff. For the purpose of this study, the analysis focused specifically on individuals diagnosed with sequelae of cerebrovascular disease, as defined by the ICD-10 classification. In this dataset, I69 was used to code documented prior stroke or post-stroke status recorded in the available medical documentation, typically after the acute phase; it was not assigned on the basis of a new clinical assessment performed within this study. Additionally, attention was given to patients with neurological conditions, particularly those with diagnoses within the G40–G62 ICD-10 range, such as epilepsy and polyneuropathy.

### 2.3. Data Analysis

In this study, descriptive statistics were used to summarize the demographic and clinical characteristics of the study population. The focus was placed on calculating frequencies, percentages, means, and medians for key variables within the total sample and selected diagnostic subgroups. Due to the exploratory nature of this study and the size of the specific diagnostic groups, no inferential statistical tests were performed.

## 3. Results

In 2023, a total of 551 homeless men resided in TPBA shelters and night shelters; the present analysis focuses on a random subsample of 226 individuals. The mean age of the analytical sample was 57.0 (SD 12.9) years (median 60). Within the total sample, 95 individuals (42.2%) held a disability certificate, 90 (40.0%) had documented substance use disorder, and 99 (44.0%) had been hospitalized since 2019. The most frequent diagnoses included essential (primary) hypertension (20.4%) (ICD-10: I10), heart failure (10.2%) (ICD-10: (I50), atrial fibrillation/flutter (8.9%) (ICD-10: I48), chronic obstructive pulmonary disease (8.4%) (ICD-10: J44.9), and sequelae of cerebrovascular disease (8.9%). Sequelae of lower limb injuries (ICD-10: T93) were also documented in 8.0% of individuals. Selected mental health diagnoses included schizophrenia (4.0%), recurrent depressive disorder (3.6%), and dementia in other diseases classified elsewhere (3.1%) (Table 1).

Among the analyzed sample, sequelae of cerebrovascular disease were identified in 20 individuals, accounting for 8.9% of the total. Compared with the overall sample, this group was older and had higher rates of disability certification and hospitalization (Table 2). The most common co-occurring conditions within this group included essential (primary) hypertension, present in 55.0%; atherosclerosis (ICD-10: I70), in 40.0%; and hemiplegia (ICD-10: G81), in 25.0% of individuals. Additionally, atrial fibrillation/flutter and heart failure were diagnosed in 25.0% and 20.0% of patients, respectively.

Among the diseases of the nervous system, epilepsy (ICD-10: G40) was diagnosed in 27 individuals (12.0%), whereas polyneuropathy (ICD-10: G62) was identified in 9 individuals (4.0%). Table 2 presents a comparison of key characteristics between these subgroups and the total sample. The G40 group was notably younger, with a mean age of 53.1 years, and had high rates of co-occurring substance use disorders (74.1%). In contrast, individuals in the G62 group were older (mean age: 62.3 years) and more frequently had a disability certificate (77.8%). The most common comorbidities in both groups included essential (primary) hypertension, atrial fibrillation/flutter, and chronic obstructive pulmonary disease, each affecting 14.8% to 33.3% of individuals. More data on the analyzed sample (including individual-level characteristics and frequency of the ICD-10 diagnoses) can be found in the Supplementary Material.

## 4. Discussion

This study is likely one of the largest medical record-based analyses of health conditions among homeless men conducted in Poland [10,14,15,16]. It includes nearly half of the estimated homeless population in Gdańsk, making the sample size substantial in relation to the overall group [13]. In contrast to many earlier studies that relied primarily on self-reported data, this research draws on verified medical documentation, offering a more objective perspective on health status among people experiencing homelessness [6,8,17].

Although the sample size may appear modest, it is comparable to other studies on homelessness in Poland and Europe, which typically include between 50 and 200 participants [10,15,16]. Our findings also appear to be consistent with broader European data. On the basis of the available data, the prevalence of major cardiovascular diseases (CVDs) included in this study was generally lower among our PEH sample than in the general Polish population [18,19,20]. CVDs are typically seen in older adults; nevertheless, their presence in this study’s relatively younger homeless cohort is striking. Notably, heart failure was an exception to this pattern and was observed in the analyzed group at a rate approximately three times higher than that reported in the Polish general population [21]. This suggests that in this cohort, CVDs are often diagnosed at a later stage than in the general population, frequently when the disease has become severe and patients are already experiencing multiple comorbidities. This may be attributed to the generally limited access to healthcare services among people experiencing homelessness [18,22,23].

The incidence of epilepsy in the sample far exceeded the prevalence of this condition in the general population, which is estimated at 1% [24]. The epilepsy subgroup in our study was also significantly younger than the overall homeless sample. Patients with epilepsy are at two to three times greater risk of premature death than the general population [25]. Further investigation is needed to determine whether idiopathic or secondary epilepsy is dominant in this group. Given the high prevalence of substance abuse in this subgroup, it is crucial to assess how often seizures due to alcohol withdrawal syndrome are misdiagnosed as epileptic seizures [26]. Conversely, PEH patients with substance use disorders are at high risk of traumatic brain injury (TBI) [27,28], which might result in posttraumatic secondary epilepsy.

There are no data on the incidence of general polyneuropathy in the Polish population. The estimated prevalence worldwide is 5–8% [29]. In our sample, individuals with polyneuropathy were older than the average individuals in the group. This is likely because peripheral neuropathies are often secondary to other conditions, most commonly long-lasting or poorly controlled diabetes or chronic alcohol abuse [29].

Compared with the housed population, PEH report worse overall health and quality of life, with a threefold greater likelihood of having chronic illness and markedly shorter life expectancy [30,31]. Numerous factors contribute to these poor health outcomes: often harsh and hostile living conditions, exposure to unpredictable weather, food insecurity, poverty, social isolation, and histories of trauma or adverse childhood experiences all negatively impact both physical and mental health [32,33]. PEH patients also have disproportionately high rates of comorbidities, which are likely related to elevated levels of alcohol and tobacco use [34]. Recent studies have shown that homeless populations bear a high burden of serious health conditions. A major meta-analysis revealed current mental health disorders in approximately 67% of homeless individuals (77% over their lifetime), far exceeding rates in the general population [35]. For example, homeless individuals have a 51% greater risk of developing neurodegenerative diseases such as Alzheimer’s disease or other dementias [33]. They also present a greater prevalence of depressive disorders [36] and schizophrenia [37] than the general population. Furthermore, cardiovascular disease is more common: one analysis from the UK reported roughly double the incidence of cardiac disease and earlier onset of cardiovascular conditions in homeless patients [34], and a systematic review revealed nearly three times greater cardiovascular morbidity and mortality in PEH populations than in healthy controls [38].

Barriers in access to primary healthcare further exacerbate these outcomes [2,39]. Homeless individuals are overrepresented among hospital patients, especially in emergency departments. A longitudinal study of hospital records in England revealed that annual hospital admission rates are 1.79 times higher for homeless adults (with emergency admissions 2.08 times higher and preventable admissions 1.65 times higher) than for housed individuals [40]. This indicates a disproportionate reliance on acute emergency care, which can lead to delayed diagnosis or conditions being detected only during crises [2]. Neglecting regular primary and preventive care not only worsens health outcomes but also generates substantial costs for the healthcare system [39,40]. Consequently, many health issues in this group are mismanaged and undertreated [39].

The combination of early-onset chronic diseases, mental illness, and substance use observed in our study participants points to the systemic neglect of PEH. Many participants presented substantial multimorbidity despite a mean age of 57 years. This finding supports the hypothesis that homelessness is a significant determinant of poor health outcomes and premature morbidity.

## 5. Limitations

This study has several limitations related to the characteristics of the data and the manner in which they were collected. Participation in services is voluntary, and outreach efforts do not reach all individuals experiencing homelessness, particularly those avoiding contact with support systems. Although the Gdańsk branch of TPBA is the largest provider of assistance for PEH in the city, it is not the only one. Consequently, some individuals may have sought help elsewhere, leading to self-selection bias in the sample. Nevertheless, the data offer valuable insight into a population that often faces significant barriers to medical care, barriers that may delay the diagnosis and treatment of neurological and vascular conditions, thereby contributing to the progression of disease and increased reliance on emergency services.

Furthermore, our analysis was limited to services primarily directed at men, which means that women and individuals of other gender identities were not represented in the dataset. This reflects broader demographic patterns; homeless populations in European countries [41], including Poland [4,9], are predominantly male. Nonetheless, it narrows the scope of our findings. The absence of female and non-male perspectives may limit the generalizability of our conclusions, particularly given potential differences in the presentation and management of neurological and vascular conditions across genders.

Additionally, the data were originally collected by staff and volunteers for administrative and support purposes rather than for scientific research. As a result, documentation practices vary and often rely on patient-reported information without medical verification. For example, it remains unclear whether individuals who reported having epilepsy were referring to a chronic neurological condition or describing isolated seizure-like events, such as those occurring during alcohol withdrawal (which would not meet the definition of an unprovoked epileptic seizure). Similarly, all stroke cases were coded by staff as I69, indicating sequelae of cerebrovascular disease, because individuals were typically admitted to shelters after experiencing a stroke rather than during the acute phase. This approach limits the ability to determine the type and severity of the stroke, the level of resulting disability, or whether the episode may have been a transient ischemic attack (TIA). Moreover, individuals who experienced severe poststroke impairment were often transferred to care institutions capable of providing a higher level of support and therefore may be underrepresented in the dataset.

## 6. Conclusions

This study provides new insight into the health status of people experiencing homelessness (PEH) in Poland, with a focus on neurological and vascular diseases. In contrast to existing research, this study is based on medical records rather than self-reports. The results revealed a high burden of chronic conditions (such as heart failure, hypertension, or sequelae of cerebrovascular disease) occurring at relatively young ages within this cohort. These findings highlight the significant burden of both physical and mental illness among people experiencing homelessness.

The coexistence of chronic neurological and cardiovascular burdens may reflect the cumulative effects of structural disadvantage, homelessness, limited access to healthcare, and the absence of preventive interventions. These outcomes highlight the need for integrated and accessible PEH healthcare and social care models. Despite limitations related to data collection and representativeness, the findings can inform targeted policies aimed at reducing health disparities and improving medical support for PEH populations.

## Figures and Tables

**Table 1:** Characteristics of the studied cohort.

Statistic	Sample (*n* = 226)
Mean age (SD)	57.0 (12.9)
Hospitalized since 2019	99 (43.8%)
Diagnosed with primary hypertension	46 (20.4%)
Diagnosed with heart failure	23 (10.2%)
Diagnosed with atrial fibrillation/flutter	20 (8.9%)
Diagnosed with COPD	19 (8.4%)
Diagnosed with schizophrenia	9 (4.0%)
Diagnosed with recurrent depressive disorder	8 (3.6%)

COPD—chronic obstructive pulmonary disease.

**Table 2:** Comparison of selected characteristics between individuals diagnosed with sequelae of cerebrovascular disease (I69), epilepsy (G40), or polyneuropathy (G62) and the total sample.

Variable	I69 Group (*n* = 20)	G40 Group (*n* = 27)	G62 Group (*n* = 9)	Total Sample (*n* = 226)
Mean age (SD)	66.3 (8.6)	53.1 (13.3)	62.3 (9.1)	57.0 (12.9)
Median age	67	50	65	60
Disability certificate	14 (70%)	17 (63.0%)	7 (77.8%)	95 (42.2%)
Treated/diagnosed with SUD	6 (30%)	20 (74.1%)	6 (66.7%)	90 (40.0%)
Mean duration of shelter stay (in days)	476.4	399.8	485.3	360.0
Hospitalized since 2019	15 (75.0%)	15 (55.6%)	8 (88.9%)	99 (44.0%)
Mean number of other diagnoses	5.6	3.9	4.4	2.4

SUD—substance use disorder.

## Data Availability

The dataset used in this study consists of retrospective medical records provided by the Saint Brother Albert’s Aid Society (TPBA). In accordance with our agreement with the organization and in order to protect the privacy and safety of the study group, full data are not publicly available. However, the individual-level characteristics (excluding age and disability certificate) and ICD-10 diagnoses frequency can be found in the Supplementary Material.

## Supplementary Materials

The following supporting information can be downloaded at https://www.mdpi.com/article/10.3390/jcm15062278/s1, Table S1. Individual-level characteristics of the analytical sample (*n* = 226), including mortality, hospitalization history, use of homelessness services, and number of documented diagnoses. Table S2. Frequency of documented ICD-10 diagnoses in the analytical sample (*n* = 226), listed by code, diagnosis name, and number of affected patients.

## Abbreviations

The following abbreviations are used in this manuscript:

CVD	Cardiovascular disease
COPD	Chronic obstructive pulmonary disease
ED	Emergency department
FEANTSA	European Federation of National Organizations Working with the Homeless (Fédération Européenne d’Associations Nationales Travaillant avec les Sans-Abri)
ICD-10	International Statistical Classification of Diseases and Related Health Problems, 10th Revision
PEH	People experiencing homelessness
SUD	Substance use disorder
TPBA	Saint Brother Albert’s Aid Society (Towarzystwo Pomocy im. Św. Brata Alberta)
TBI	Traumatic brain injury
TIA	Transient ischemic attack
