# Supplementary material for: Health Challenges in Vulnerable Populations: Neurological and Vascular Diseases Among People Experiencing Homelessness in Gdańsk, Poland: An Observational Study
Source: J Clin Med. 2026 Mar 17;15(6):2278. doi: 10.3390/jcm15062278 (PMC13027047; doi:10.3390/jcm15062278)
Supplement: Supplementary file 1 [file jcm-15-02278-s001.zip › jcm-4152781-supplementary.pdf]

Table S1. Individual-level characteristics of the analytical sample (n = 226), including mortality, hospitalization history, use of homelessness services, and number of documented diagnoses.

DBA – Brother Albert's House (Dom Brata Alberta)

	**Death in 2023?**	**Reported hospitalization since 2019?**	**Number of hospitalizations in 2023**	**Number of night-shelter stays**	**Night-shelter days**	**Number of shelter stays**	**Shelter days**	**Number of DBA stays**	**DBA days**	**Number of diagnoses**
0	0	1	1	5	311	0	0	0	0	6
1	0	0	0	1	365	0	0	0	0	0
2	0	0	0	0	0	1	365	0	0	2
3	0	1	2	0	0	0	0	1	231	5
4	0	0	0	2	17	1	244	0	0	1
5	0	0	0	1	11	0	0	0	0	1
6	0	0	0	0	0	1	365	0	0	1
7	0	0	0	1	22	1	1	0	0	4
8	0	1	3	0	0	3	163	0	0	10
10	0	1	2	14	114	1	15	0	0	7
11	0	1	0	1	365	0	0	0	0	0
12	0	0	0	2	101	0	0	0	0	0
13	0	1	3	0	0	0	0	1	354	8
14	0	1	1	0	0	2	169	1	193	7
15	0	0	0	1	11	0	0	0	0	0
16	0	1	1	1	15	1	164	1	89	3
17	0	0	0	0	0	0	0	1	365	2
18	0	0	0	2	2	0	0	0	0	0
19	0	0	0	3	68	0	0	0	0	1
20	0	0	0	0	0	0	0	1	365	3
21	1	0	0	1	210	1	80	0	0	2
22	0	0	0	2	23	0	0	0	0	0
23	0	0	0	2	206	0	0	0	0	3
24	0	0	1	4	26	2	68	1	178	3
25	0	0	0	0	0	2	348	0	0	1
26	0	1	1	0	0	0	0	1	186	10
27	0	1	1	0	0	0	0	1	95	2
28	0	0	0	0	0	0	0	1	73	0
29	0	0	0	0	0	1	365	0	0	1
30	0	1	1	0	0	0	0	2	291	5
31	0	1	0	1	73	2	288	0	0	7
32	1	0	0	0	0	0	0	1	365	3
33	0	1	1	0	0	0	0	1	45	1
34	0	1	2	0	0	0	0	1	365	12
35	0	0	0	0	0	0	0	1	365	1

36	0	0	0	4	36	0	0	0	0	0
37	0	0	0	1	68	0	0	0	0	0
38	0	1	1	0	0	0	0	1	244	5
39	0	0	0	0	0	1	365	0	0	3
40	0	1	1	0	0	0	0	1	328	2
41	0	0	0	0	0	0	0	1	75	0
42	0	0	0	1	336	0	0	0	0	0
43	0	0	0	1	5	0	0	0	0	0
44	0	1	1	0	0	0	0	1	56	1
45	0	0	0	1	74	0	0	0	0	4
46	0	1	1	0	0	0	0	1	350	4
47	0	0	0	1	5	0	0	1	58	3
48	0	1	0	0	0	0	0	2	338	2
49	0	0	0	0	0	0	0	1	220	2
50	0	0	0	2	33	0	0	0	0	0
51	0	0	0	0	0	1	12	0	0	3
52	0	0	0	6	211	0	0	0	0	0
53	0	1	0	1	278	1	87	0	0	3
54	0	0	0	0	0	0	0	1	12	3
55	0	0	0	0	0	1	365	0	0	0
56	0	1	3	0	0	1	192	0	0	7
57	0	0	0	0	0	0	0	1	365	3
58	0	1	0	8	134	0	0	0	0	3
59	0	1	1	1	5	3	179	1	63	6
60	0	0	0	0	0	0	0	1	11	0
61	0	1	1	2	16	0	0	0	0	1
62	0	1	1	0	0	0	0	1	20	0
63	0	0	0	2	114	0	0	0	0	0
64	0	0	0	0	0	0	0	1	365	2
65	0	0	0	0	0	1	35	0	0	1
66	0	0	0	0	0	1	40	0	0	1
67	0	1	1	0	0	0	0	1	26	1
68	0	0	0	0	0	0	0	1	27	0
69	0	0	0	2	5	0	0	0	0	0
70	0	0	0	0	0	1	365	0	0	1
71	0	0	0	0	0	1	365	0	0	1
72	0	0	0	0	0	1	365	0	0	4
73	0	1	1	0	0	1	297	0	0	2
74	0	0	0	2	150	0	0	0	0	1
75	0	1	11	0	0	3	338	0	0	11
76	0	1	1	0	0	0	0	1	2	5
77	0	0	0	2	51	0	0	0	0	0
78	0	1	1	2	107	2	254	0	0	3
79	0	0	0	1	365	0	0	0	0	1
80	0	0	0	2	112	0	0	0	0	1
81	0	1	4	0	0	1	250	0	0	3

82	0	1	0	0	0	0	0	1	365	5
83	0	0	0	0	0	1	365	0	0	0
84	0	0	0	1	2	0	0	0	0	3
85	0	0	0	0	0	1	365	0	0	0
86	1	1	1	0	0	0	0	1	4	2
87	0	1	2	0	0	0	0	1	210	8
88	0	0	0	1	4	0	0	0	0	2
89	0	0	0	3	12	0	0	0	0	2
90	0	0	0	1	1	0	0	0	0	0
91	0	0	0	2	62	0	0	0	0	1
92	0	1	0	0	0	0	0	1	365	3
93	0	1	0	1	4	1	75	0	0	3
94	0	0	0	1	9	0	0	0	0	0
95	0	1	1	0	0	1	365	0	0	7
96	0	0	0	6	287	0	0	0	0	4
97	0	0	0	1	231	0	0	0	0	0
98	0	0	0	0	0	1	365	0	0	1
99	0	0	0	0	0	0	0	1	365	3
100	0	0	0	1	1	0	0	0	0	0
101	0	1	1	0	0	2	333	0	0	4
102	0	1	0	1	2	0	0	0	0	2
103	0	1	1	0	0	0	0	2	141	8
104	0	1	0	0	0	0	0	1	365	3
105	0	0	0	0	0	3	295	0	0	0
106	0	1	1	1	108	1	12	0	0	0
107	0	0	0	3	67	0	0	0	0	1
108	0	0	0	7	336	0	0	0	0	0
109	0	1	2	0	0	0	0	1	135	1
110	0	0	0	2	41	0	0	0	0	1
111	0	0	0	2	30	0	0	0	0	0
112	0	1	3	0	0	2	352	0	0	1
113	0	0	0	1	365	0	0	0	0	0
114	0	0	0	4	27	3	126	0	0	1
115	0	1	0	1	5	0	0	0	0	2
116	0	0	0	0	0	0	0	1	365	4
117	0	0	0	2	291	1	72	0	0	2
118	0	1	3	3	31	0	0	4	188	2
119	0	0	0	0	0	1	365	0	0	3
120	0	0	0	1	306	0	0	0	0	0
121	0	1	3	0	0	0	0	1	152	3
122	0	0	0	0	0	1	178	0	0	2
123	0	0	0	1	24	0	0	0	0	0
124	0	0	0	0	0	1	22	0	0	0
125	0	0	0	0	0	2	365	0	0	1
126	0	0	0	1	1	0	0	0	0	0
127	0	1	3	0	0	0	0	1	81	5

128	0	1	1	11	197	0	0	0	0	2
129	0	0	0	1	365	0	0	0	0	0
130	0	1	4	0	0	0	0	1	59	6
131	0	0	0	0	0	0	0	1	25	0
132	0	1	1	0	0	1	14	0	0	6
133	0	1	0	1	365	0	0	0	0	1
134	0	1	1	0	0	1	365	0	0	9
135	0	1	2	0	0	0	0	1	365	6
136	0	1	0	0	0	0	0	1	365	7
137	0	1	2	8	87	3	158	0	0	2
138	0	0	0	8	191	0	0	0	0	1
139	0	0	0	0	0	1	365	0	0	3
140	0	1	0	0	0	1	365	0	0	4
141	0	1	0	4	101	1	136	0	0	3
142	0	1	3	0	0	0	0	2	193	5
143	0	0	0	1	11	0	0	0	0	0
144	0	1	0	2	55	0	0	0	0	1
145	0	0	0	4	126	0	0	0	0	1
146	0	1	0	0	0	1	90	0	0	4
147	0	1	1	0	0	0	0	1	365	9
148	0	1	0	1	58	1	268	1	38	2
149	0	1	2	0	0	0	0	1	14	5
150	1	0	0	0	0	0	0	1	107	7
151	0	1	1	0	0	0	0	2	351	6
152	0	0	0	1	365	0	0	0	0	1
153	0	1	1	0	0	0	0	2	319	1
154	1	0	0	5	137	0	0	0	0	2
155	0	0	0	1	306	0	0	0	0	0
156	0	0	0	0	0	1	1	0	0	1
157	0	1	0	0	0	0	0	1	32	0
158	0	0	0	2	8	0	0	0	0	0
159	0	1	2	0	0	2	47	4	91	3
160	0	0	0	2	57	0	0	0	0	0
161	0	1	1	9	334	0	0	0	0	5
162	0	0	0	1	6	0	0	0	0	1
163	0	0	1	4	336	0	0	0	0	0
164	0	1	0	0	0	0	0	1	365	3
165	0	1	1	0	0	1	298	0	0	5
166	0	0	0	0	0	1	136	0	0	4
167	0	0	0	0	0	0	0	1	32	0
168	0	1	0	0	0	0	0	1	365	1
169	0	1	1	0	0	1	28	3	242	5
170	0	1	0	1	333	0	0	0	0	2
171	0	1	1	0	0	0	0	1	62	0
172	0	0	0	2	245	0	0	0	0	1
173	0	0	0	0	0	0	0	2	251	1

174	0	0	0	1	3	0	0	0	0	0
175	0	0	0	0	0	1	1	0	0	3
176	0	0	0	0	0	0	0	1	365	3
177	0	0	0	3	100	0	0	0	0	0
178	0	1	1	0	0	0	0	1	365	6
179	0	0	0	0	0	2	352	0	0	1
180	0	0	0	0	0	0	0	1	123	2
181	0	0	0	1	11	0	0	0	0	0
182	0	0	0	0	0	1	26	3	332	4
183	0	1	4	0	0	1	365	0	0	2
184	0	1	1	3	72	0	0	2	129	7
185	0	1	0	0	0	1	365	0	0	3
186	0	1	1	1	18	0	0	2	265	10
187	0	1	3	0	0	0	0	3	255	10
188	0	0	0	0	0	0	0	1	365	1
189	0	0	0	1	68	0	0	0	0	3
190	0	1	2	0	0	0	0	2	95	4
191	0	1	0	1	46	2	190	0	0	4
192	0	0	0	0	0	0	0	1	298	1
193	0	0	0	0	0	0	0	1	365	3
194	0	0	0	8	179	0	0	0	0	1
195	0	0	0	0	0	0	0	1	1	4
196	1	0	0	1	14	0	0	0	0	0
197	0	0	0	2	354	1	11	0	0	0
198	0	0	0	1	365	0	0	0	0	0
199	0	1	1	0	0	0	0	1	60	2
200	0	1	1	0	0	0	0	1	321	4
201	1	1	0	0	0	0	0	1	184	0
202	0	0	0	0	0	1	365	0	0	2
203	0	1	3	8	287	0	0	0	0	2
204	0	1	0	0	0	0	0	1	365	2
205	0	0	0	2	227	0	0	0	0	3
206	0	1	0	0	0	0	0	1	260	2
207	0	0	0	1	365	0	0	0	0	1
208	0	1	0	0	0	0	0	1	9	1
209	0	1	0	0	0	0	0	1	365	1
210	0	1	0	0	0	1	365	0	0	0
211	0	1	2	0	0	1	44	1	294	3
212	0	0	0	3	349	0	0	0	0	0
213	0	0	0	6	317	0	0	0	0	3
214	0	0	0	0	0	1	365	0	0	2
215	0	0	0	6	49	0	0	0	0	1
216	0	1	4	0	0	1	365	0	0	5
217	0	1	5	2	8	3	160	2	58	4
218	0	1	1	12	244	0	0	0	0	0
219	0	0	0	5	110	0	0	0	0	0

220	0	1	2	1	33	2	329	0	0	5
221	0	1	3	1	73	1	75	0	0	1
222	0	1	2	0	0	0	0	1	5	3
223	0	0	0	1	27	0	0	0	0	0
224	0	0	0	0	0	0	0	1	33	0
225	0	0	0	1	9	0	0	0	0	0

Table S2. Frequency of documented ICD-10 diagnoses in the analytical sample (n = 226), listed by code, diagnosis name, and number of affected patients.

ICD-10 – International Classification of Diseases, 10th Revision

ICD-10 Code	Disease Name	Number of Patients
I10	Essential (primary) hypertension	46
G40	Epilepsy	27
I50	Heart failure	23
I48	Atrial fibrillation and flutter	20
I69	Sequelae of cerebrovascular disease	20
J44.9	Chronic obstructive pulmonary disease, unspecified	19
T93	Sequelae of injuries of lower limb	18
I70	Atherosclerosis	17
M47	Spondylosis	12
T91	Sequelae of injuries of neck and trunk	12
T90	Sequelae of injuries of head	11
T92	Sequelae of injuries of upper limb	11
E78	Disorders of lipoprotein metabolism and other lipidaemias	10
K40	Inguinal hernia	10
F20	Schizophrenia	9
G62	Other polyneuropathies	9
I73.9	Peripheral vascular disease, unspecified	9
J45	Asthma	9
F33	Recurrent depressive disorder	8
H25	Senile cataract	8
I25.2	Old myocardial infarction	8
I26	Pulmonary embolism	8
Z95	Presence of cardiac and vascular implants and grafts	8
F02	Dementia in other diseases classified elsewhere	7
I25	Chronic ischaemic heart disease	7
N40	Hyperplasia of prostate	7
B18	Chronic viral hepatitis	6
C34	Malignant neoplasm of bronchus and lung	6
E10	Type 1 diabetes mellitus	6
E11	Type 2 diabetes mellitus	5
F07	Personality and behavioural disorders due to brain disease, damage and dysfunction	5
F19	Mental and behavioural disorders due to multiple drug use and use of other psychoactive substances	5
G81	Hemiplegia	5

H90	Conductive and sensorineural hearing loss	5
K21	Gastro-oesophageal reflux disease	5
K74	Fibrosis and cirrhosis of liver	5
K86	Other diseases of pancreas	5
M54.5	Low back pain	5
N20	Calculus of kidney and ureter	5
F78	Other mental retardation	4
I42	Cardiomyopathy	4
I71	Aortic aneurysm and dissection	4
K25	Gastric ulcer	4
K26	Duodenal ulcer	4
K70	Alcoholic liver disease	4
K71	Toxic liver disease	4
A15	Respiratory tuberculosis, bacteriologically and histologically confirmed	3
C61	Malignant neoplasm of prostate	3
E66	Obesity	3
H40	Glaucoma	3
L40	Psoriasis	3
M10	Gout	3
M16	Coxarthrosis [arthrosis of hip]	3
B20	Human immunodeficiency virus [HIV] disease resulting in infectious and parasitic diseases	2
D18	Haemangioma and lymphangioma, any site	2
E03	Other hypothyroidism	2
E33	Disorders of other endocrine glands	2
G45	Transient cerebral ischaemic attacks and related syndromes	2
G92	Toxic encephalopathy	2
I35.1	Aortic (valve) insufficiency	2
I83.0	Varicose veins of lower extremities with ulcer	2
K80	Cholelithiasis	2
M54.3	Sciatica	2
M72.0	Palmar fascial fibromatosis [Dupuytren]	2
N18	Chronic kidney disease	2
Z89.8	Acquired absence of other limb(s)	2
A41	Other septicaemia	1
A69.2	Lyme disease	1
C01	Malignant neoplasm of base of tongue	1
C08	Malignant neoplasm of other and unspecified major salivary glands	1
C16	Malignant neoplasm of stomach	1
C18	Malignant neoplasm of colon	1
C19	Malignant neoplasm of rectosigmoid junction	1
C25	Malignant neoplasm of pancreas	1
C26	Malignant neoplasm of other and ill-defined digestive organs	1
C71	Malignant neoplasm of brain	1
C92	Myeloid leukaemia	1

E79.0	Hyperuricaemia without signs of inflammatory arthritis and tophaceous disease	1
E81	Disorders of mineral metabolism	1
F00	Dementia in Alzheimer's disease	1
F11	Mental and behavioural disorders due to use of opioids	1
F13	Mental and behavioural disorders due to use of sedatives or hypnotics	1
F31	Bipolar affective disorder	1
F41	Other anxiety disorders	1
F92	Mixed disorders of conduct and emotions	1
G12.2	Motor neuron disease	1
G54	Nerve root and plexus disorders	1
G82.2	Paraplegia, unspecified	1
I33	Acute and subacute endocarditis	1
I34	Nonrheumatic mitral valve disorders	1
I34.0	Mitral (valve) insufficiency	1
I49.8	Other specified cardiac arrhythmias	1
I70.2	Atherosclerosis of arteries of extremities	1
I85	Oesophageal varices	1
I87.2	Venous insufficiency (chronic) (peripheral)	1
J47	Bronchiectasis	1
J93	Pneumothorax	1
K20	Oesophagitis	1
K43	Ventral hernia	1
K44	Diaphragmatic hernia	1
K76.0	Fatty (change of) liver, not elsewhere classified	1
K85	Acute pancreatitis	1
M17	Gonarthrosis [arthrosis of knee]	1
M79.7	Fibromyalgia	1
M81	Osteoporosis without pathological fracture	1
N17	Acute renal failure	1
N39.0	Urinary tract infection, site not specified	1
R64	Cachexia	1
X84	Intentional self-harm by unspecified means	1
Z93.3	Colostomy status	1
